# Kaposi’s sarcoma-associated herpesvirus ORF34 is essential for late gene expression and virus production

**DOI:** 10.1038/s41598-017-00401-7

**Published:** 2017-03-23

**Authors:** Mayu Nishimura, Tadashi Watanabe, Syota Yagi, Takahiro Yamanaka, Masahiro Fujimuro

**Affiliations:** Department of Cell Biology, Kyoto Pharmaceutical University, Misasagi-Shichono-cho 1, Yamashina-ku, Kyoto 607-8412 Japan

## Abstract

Kaposi’s sarcoma-associated herpesvirus (KSHV) is the causative agent of Kaposi’s sarcoma, primary effusion lymphoma, and multicentric Castleman’s disease. KSHV establishes a life-long infection in its host and alternates between a latent and lytic infection state. During lytic infection, lytic-related genes are expressed in a temporal manner and categorized as immediate early, early, and late gene transcripts. ORF34 is an early-late gene that interacts with several viral transcription-associated factors, however its physiological importance remains poorly understood. Here, we investigated the role of ORF34 during KSHV infection by generating ORF34-deficient KSHV, using a bacterial artificial chromosome system. Our results reveal that ORF34-deficient KSHV exhibited significantly attenuated late gene expression and viral production but did not affect viral DNA replication. ORF34 interacted with transcription factors ORF18, ORF24, ORF31, and ORF66, and a novel ORF34-interaction partner, ORF23. The C-terminal region of ORF34 was important for interaction with ORF24 and viral production. Our data support a model, in which ORF34 serves as a hub for recruiting a viral transcription complex to ORF24 to promote late viral gene expression.

## Introduction

Kaposi’s sarcoma-associated herpesvirus (KSHV) belongs to the gamma–herpesvirus family. It was first discovered in a Kaposi’s sarcoma lesion of an AIDS patient in 1994^1^, and is also known as human herpesvirus 8 (HHV-8). KSHV is closely associated with an endothelial cell malignancy, Kaposi’s sarcoma, and B-cell malignancies, primary effusion lymphoma (PEL), and multicentric Castleman’s disease^2–4^. The neoplastic potential of KSHV has been well established, especially within the context of immunosuppressed patients who are undergoing organ transplant or co-infected with HIV-1^1–5^.

Like other herpesviruses, KSHV establishes a life-long infection in its host and exists in either a latent or lytic state. During latent infection^6^, the KSHV genome circularizes to form an episome in the nucleus, leading to the expression of several latent associated genes (including LANA, v-FLIP, Kaposin, and microRNAs) that affect cell proliferation and apoptosis, and contribute to KSHV-associated malignancies^7–11^. Upon reactivation, lytic-related genes are tightly regulated in a temporal and sequential manner, which can be divided into three transcriptional stages: immediate early (IE), early (E), and late (L)^12, 13^. The alternation of KSHV between lytic replication and latency depends on the IE-gene RTA/ORF50, which triggers transcriptional activation of E genes associated with viral DNA replication^13, 14^. Transcripts of E genes initiate DNA replication from the *Oli*Lyt site of the KSHV genome and transcription of L genes encoding structural and functional proteins for producing viral particles^15^. The regulation of IE and E gene transcription has been extensively investigated, whereas the precise transcriptional machinery controlling the expression of L genes is still unknown.

In the case of EBV, a gamma-herpesvirus, the transcriptional initiation of L genes requires the formation of a transcription complex termed the virus specific pre-initiation complex (vPIC) consisting of at least 6 transcription factors^16^. BcRF1, a core factor of EBV-PIC and virus homolog of TATA box binding protein (TBP), is essential for L gene expression^17^. BcRF1 interacts with RNA polymerase II (RNAPII)^16^ and a non-canonical TATA box sequence^18, 19^, TATT. In addition, two factors, BFRF2 and BDLF4, are essential for the transcription of EBV L genes^16, 20^. While the machinery involved in the initiation of L gene transcripts of EBV have been reported, the components of KSHV-PIC have yet to be elucidated.

KSHV open reading frames (ORFs) 18, 24, 30, 31, 34, and 66, correspond with EBV-PIC components BVLF1, BcRF1, BDLF3.5, BDLF4, BGLF3, and BFRF2, respectively^16^. With the exception of ORFs 34 and 66, the other KSHV ORFs have been investigated using specific ORF-deficient recombinant KSHVs, generated by a bacterial artificial chromosome (BAC) system. ORF18^21^, ORF24^22, 23^, ORF30^21^, ORF31^24^ are critical transcription factors for L gene expression but not for IE or E gene expression. KSHV ORF24 (a homolog of EBV BcRF1) binds to RNAPII^22^, ORF34 protein^23^, and TATT sequences in the L gene promoter (K8.1 and ORF57 promoter)^22, 24^. ORF34 bridges the interaction between ORF24 and ORF31, and the ORF31-ORF34 interaction is essential for L gene expression^24^. A split luciferase assay revealed that ORF34 may also physically interact with ORFs 18, 31, and 66. Furthermore the ORF24-ORF34 interaction is essential for the expression of L genes^23^. Although ORF34 appears to serve as a scaffold for viral TBP ORF24 and PIC components (ORF18, 30, 31, 34, and 66), its functional role during KSHV replication remains unknown. Here, we generated ORF34-deficient KSHV using a BAC system and evaluated its role during viral replication. We show that ORF34 binds with ORF23, a novel ORF34-interaction partner, and regulates KSHV L gene expression.

## Results

### Mutagenesis of KSHV ORF34

**Figure 1:**
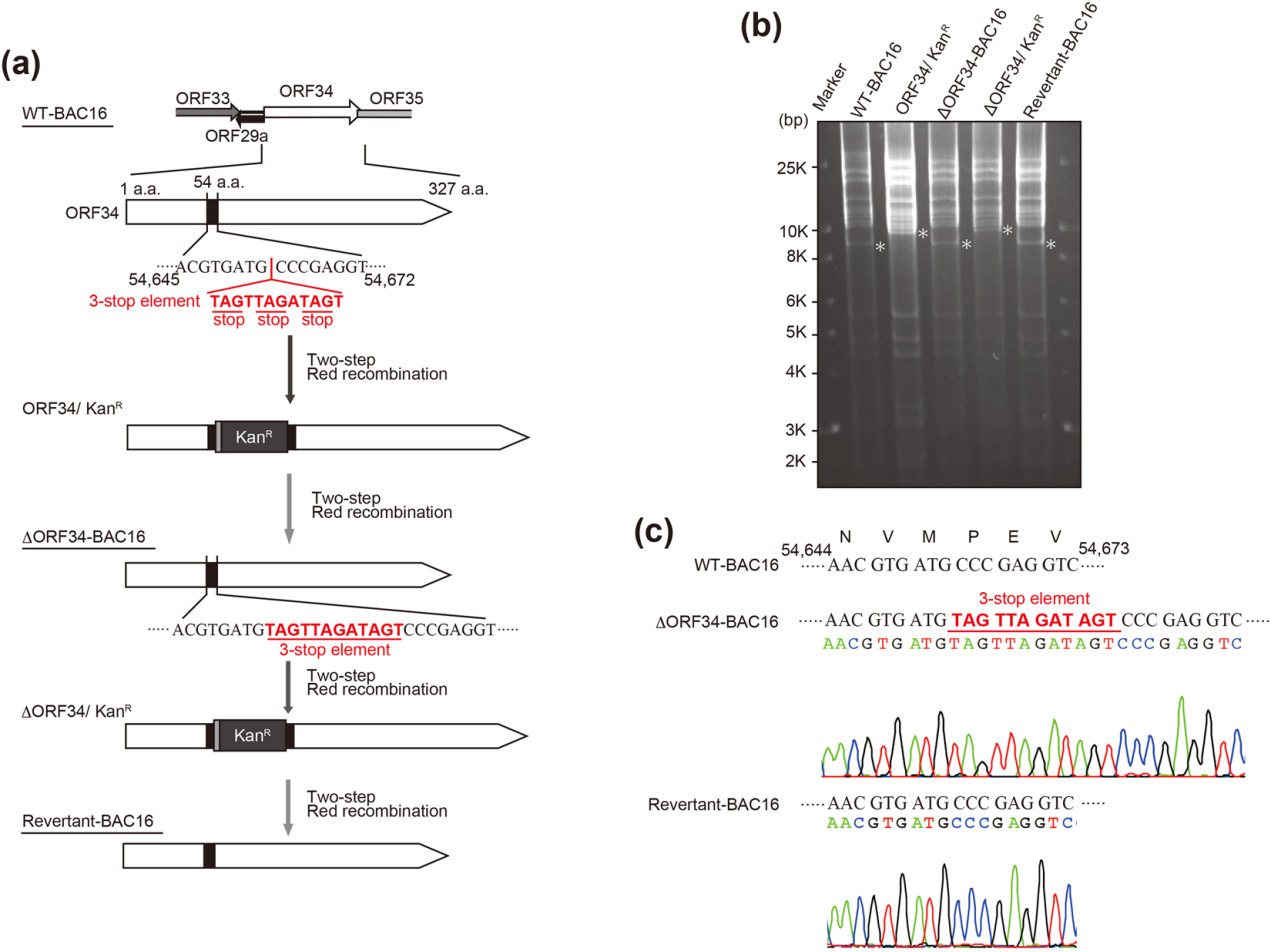
Construction of recombinant ΔORF34 KSHV BAC and its revertant. (**a**) Schematic illustration of the KSHV genome including the ORF34 coding region. Using a two-step Red recombination system, three stop codons were inserted into the ORF34 coding region of KSHV BAC16 (nt54653 – nt54654; Accession number: GQ994935) to construct ORF34-deficient BAC clone (ΔORF34-BAC16). The stop codons were replaced with the original wild-type sequence for revertant BAC clone (Revertant-BAC16). (**b**) Agarose-gel electrophoresis of the recombinant KSHV BACmids, digested with EcoRI. The asterisks (*) indicate Insertion and deletion of a kanamycin-resistance cassette in each BAC clone. Original data is shown in Supplementary Fig. S1. (**c**) DNA sequencing results of ORF34 mutagenesis sites in ΔORF34-BAC16 and Revertant-BAC16.

To elucidate the role of ORF34 during KSHV replication, we constructed ORF34-deficient recombinant KSHV BAC (ΔORF34-BAC16) and its revertant (Revertant-BAC16). A sequence of three stop codons (3-stop element) was inserted into the ORF34 coding region of KSHV BAC16 using a two-step markerless red recombination system^25, 26^ (Fig. 1a). Because the ORF34 gene overlaps with flanking ORFs (Fig. 1a), the 3-stop element was inserted within ORF34 to avoid interference with the promoter region responsible for the expression of ORFs 35–37, as previously reported^27–29^. The insertion and deletion of a kanamycin resistance cassette (Kan^R^) were confirmed by restriction enzyme (EcoRI) digestion and agarose gel electrophoresis (Fig. 1b). Sequences of mutations, the insertion of the 3-stop element for ΔORF34-BAC16 and reversion to the WT sequence for Revertant-BAC16 were confirmed by Sanger sequencing (Fig. 1c).

### ORF34 deficiency decreases virus production and late gene expression

To obtain efficient KSHV virus-producing cells, we generated tetracycline/doxycycline-inducible (Tet-on) RTA/ORF50-expressing Vero (iVero) cells. Recombinant KSHV BAC clones (wild type (WT)-BAC16, ΔORF34-BAC16, Revertant-BAC16) were transfected into iVero cells and selected with puromycin and hygromycin. We then established three recombinant KSHV-inducible stable cell lines, iVero-WT, iVero-ΔORF34, and iVero-Revertant harboring WT-BAC16, ΔORF34-BAC16, and Revertant-BAC16, respectively. These cells were treated with doxycycline (Dox) and sodium butyrate (NaB) to induce RTA expression, leading to the induction of the lytic gene expression and subsequent production of recombinant KSHV progeny virions. The recombinant KSHV virions (termed WT-KSHV, ΔORF34-KSHV and Revertant-KSHV) were isolated from media and used for further experiments.

**Figure 2:**
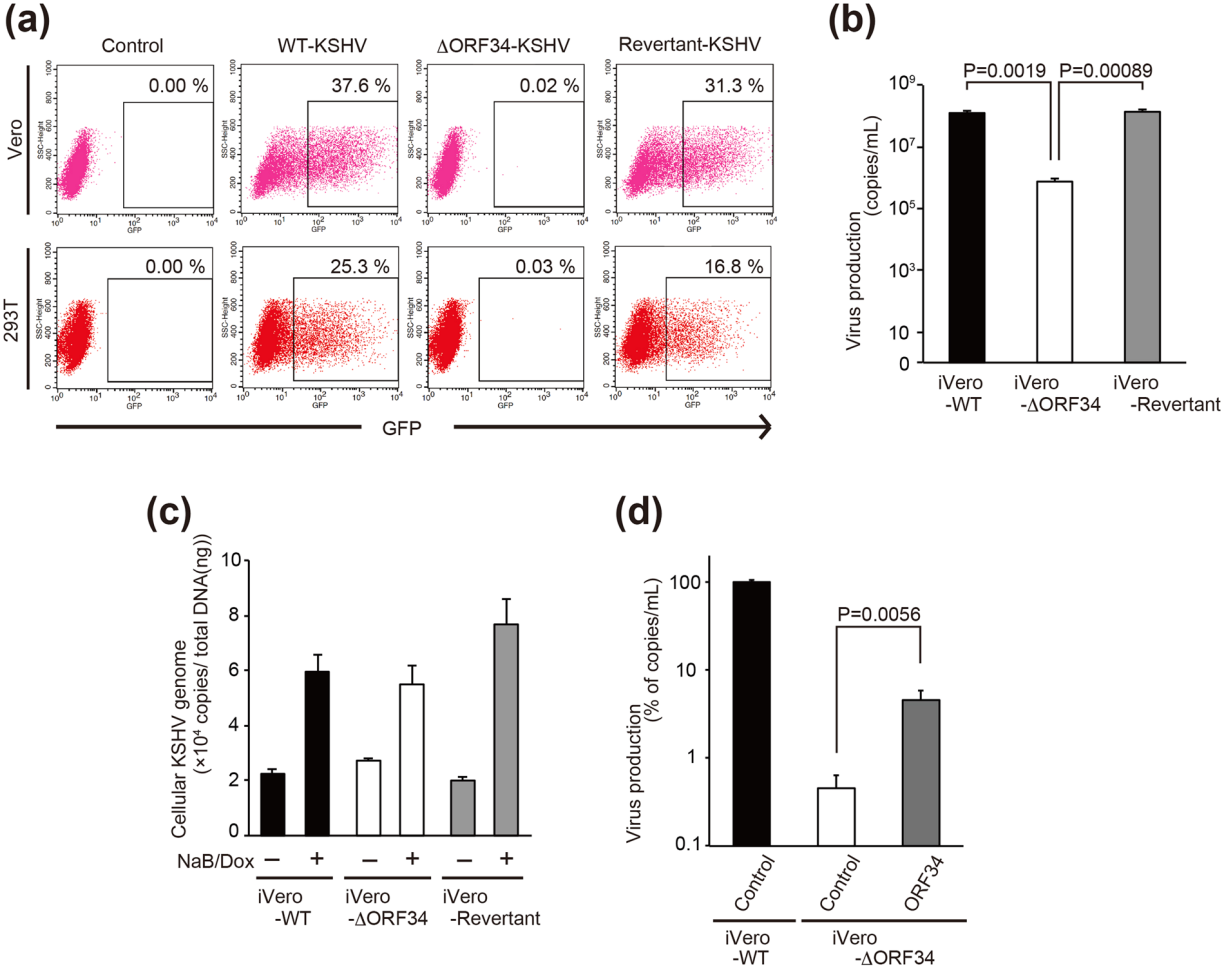
ORF34 is essential for virus production but not DNA replication of KSHV. (**a**) The effects of ORF34 deficiency on recombinant KSHV production. Each iVero cell line (iVero-WT, iVero-ΔORF34, or iVero-Revertant) were treated with Dox and NaB to induce the lytic-cycle and viral production, and cultured media was harvested after 96 h. Viral precipitates were inoculated onto cells (Vero and 293T) and infection with each KSHV was carried out for 48 h. GFP positive cells were analyzed by flow-cytometer to determine the infectivity of the recombinant viruses. The forward scatter/side scatter plots and gates of Fig. 2a are shown in Supplementary Fig. S2. (**b**) Virus production in iVero-WT, iVero-ΔORF34, iVero-Revertant. Each iVero cell line was cultured for 48 h with medium containing of NaB and Dox. KSHV DNA were purified from capsidated KSHV virions in culture supernatants, and KSHV genome copies were determined by real-time PCR. (**c**) KSHV DNA replication in iVero-WT, iVero-ΔORF34, and iVero-Revertant cells. The iVero cells were cultured for 48 h with medium containing of NaB and Dox. Cellular genome containing KSHV genome DNA were purified from each cell lines. KSHV genome copies were determined by real-time PCR and normalized by the total DNA amount. (**d**) Rescue of virus production in iVero-ΔORF34 cells by exogenous ORF34 expression. The iVero-WT or iVero-ΔORF34 cells were transfected with control plasmid or ORF34 plasmids. After 2 days, transfected cells were cultured with NaB and Dox-containing medium for 3 days, and culture supernatant containing virus was harvested. The KSHV genome was quantified by real-time PCR. (**b**–**d**) Three or four independent samples were evaluated by real-time PCR. The error bars indicate standard deviations.

To assess whether ORF34 is critical for virus production, the function of ORF34 during virus production was evaluated. iVero-WT, iVero-ΔORF34, or iVero-Revertant cells were treated with Dox and NaB, and culture supernatants were harvested. The levels of recombinant viruses (WT-KSHV, ΔORF34-KSHV, or Revertant-KSHV) contained in each culture media were measured by an infection assay (Fig. 2a) and real-time PCR (Fig. 2b). Virus particles were pelleted by ultra-centrifugation from equal amounts of cultured media, resuspended in fresh media and then inoculated onto Vero or 293 T cells for analysis. Viruses from the media of iVero-WT and iVero-Revertant cells infected approximately 38% and 31% of Vero cells, and 25% and 17% of 293 T, respectively (Fig. 2a). In contrast, infection in either cell line was not observed upon inoculation of virus concentrated from iVero-ΔORF34 cells. In agreement with these results, real-time PCR revealed that the production of WT-KSHV and Revertant-KSHV were nearly equal (Fig. 2b), while the production of the ΔORF34-KSHV was about 100-fold lower. Interestingly, there were no differences in KSHV DNA levels among the KSHV producer cell lines (Fig. 2c). To clarify the role of ORF34 during replication, we elucidated whether exogenous ORF34 expression could rescue the virus production. When iVero-ΔORF34 cells were transfected with an ORF34 expression plasmid, viral production was partially but significantly recovered, compared with those in empty plasmid-transfected cells (Fig. 2d).

**Figure 3:**
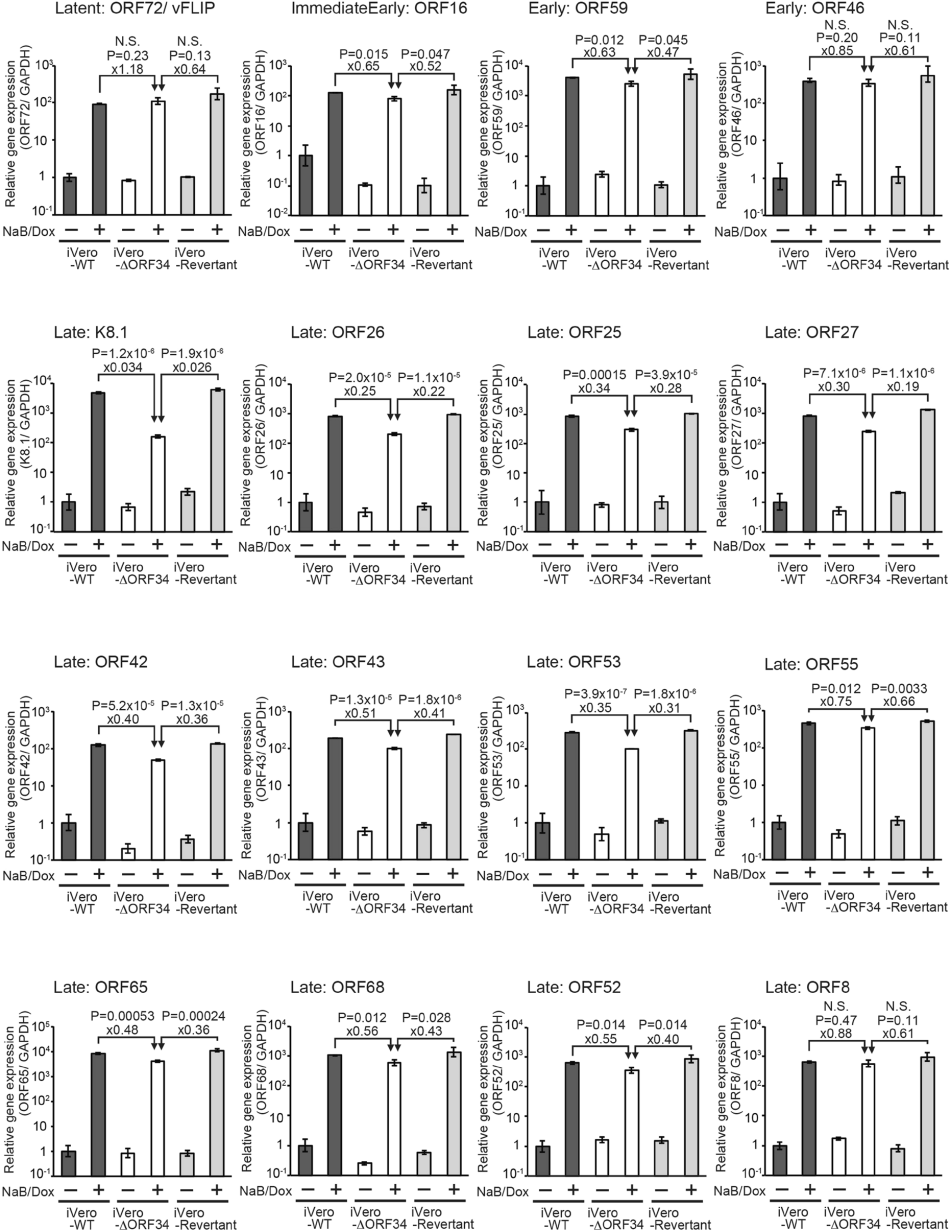
ORF34 is required for the late gene expression but not for latent, IE, and E genes expression. The iVero-WT, iVero-ΔORF34, and iVero-Revertant cells were cultured in media with/or without NaB and Dox to induce a lytic state. Total RNA was extracted from cells and subjected to RT-qPCR. The mRNA expression levels of viral genes, latent: ORF72 (v-cyclin); IE: ORF16 (vBcl-2); E: ORF46 (uracil DNA glycosylase) and ORF59 (viral DNA polymerase processivity factor); L: K8.1 (glycoprotein), ORF26 (capsid protein), ORF25 (capsid protein), ORF27 (putative glycoprotein), ORF42 (unknown tegument protein), ORF43 (capsid protein), ORF53 (glycoprotein), ORF55 (unknown tegument protein), ORF65 (capsid protein), ORF68 (glycoprotein), ORF52 (capsid protein), and ORF8 (glycoprotein), were normalized by GAPDH expression. Expression levels were assessed using three independent samples, and error bars indicate ± standard deviations.

Since ORF34 is essential for infectivity and virus production of KSHV, we next elucidated the effects of the ORF34-defect on the expression of latent and lytic genes. The mRNA expression of latent (ORF72), IE (ORF16), E (ORF46 and ORF59), and L (K8.1, ORF26, ORF25, ORF27, ORF42, ORF43, ORF53, ORF55, ORF65, ORF68, ORF52, ORF8) genes was evaluated by RT real-time PCR in induced iVero-WT, iVero-ΔORF34, and iVero-Revertant cells. Expression of latent, IE, E genes were comparable among iVero-WT, iVero-ΔORF34, and iVero-Revertant cells. In contrast, expression of L genes were remarkably attenuated in iVero-ΔORF34 cells. In particular, K8.1 expression was about 100-fold lower in iVero-ΔORF34 cells (Fig. 3).

### Physical interaction between ORF34 and KSHV-PIC components, ORF24, ORF31, ORF18, ORF23, and ORF66

**Figure 4:**
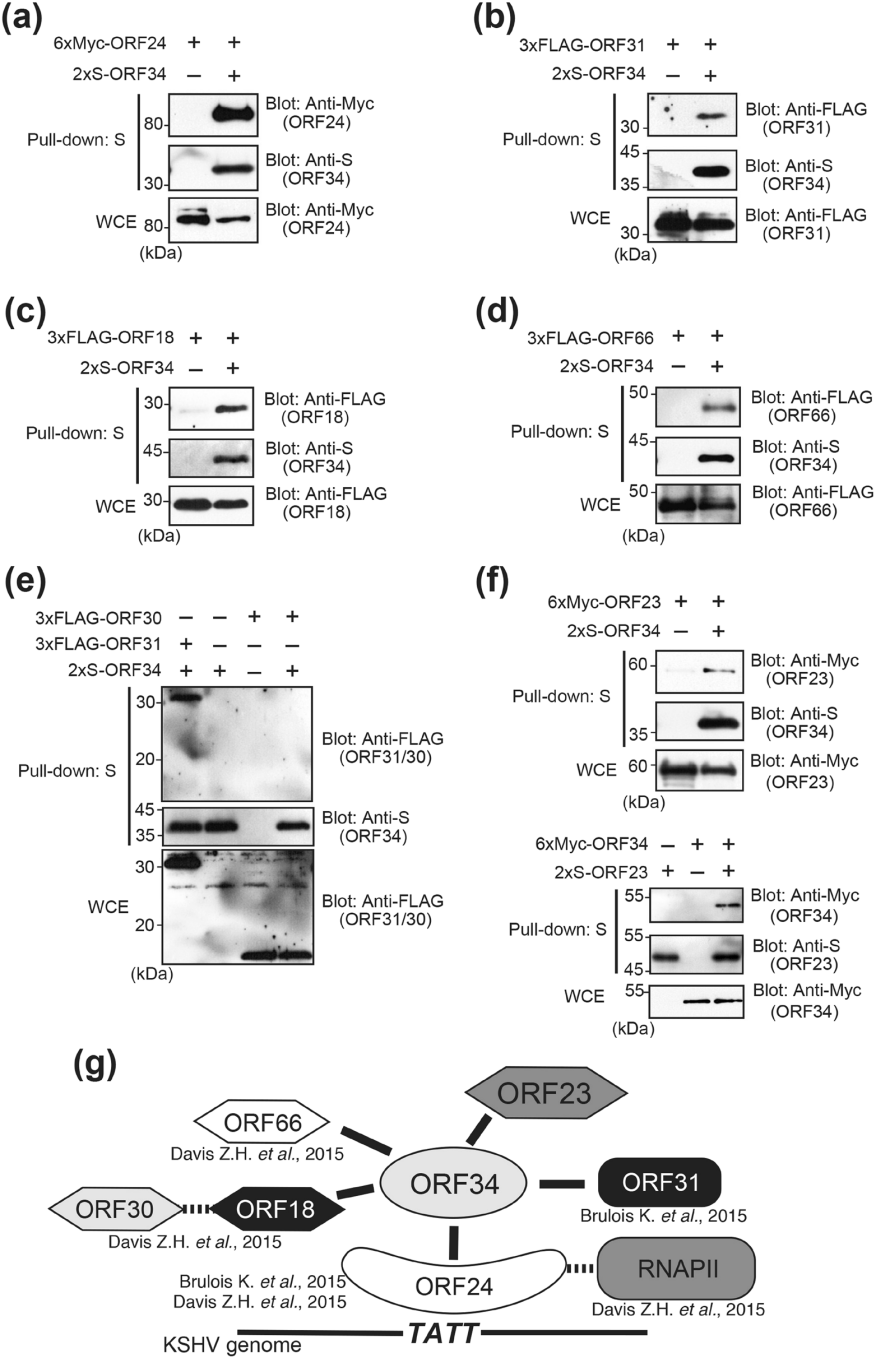
ORF34 physically interacts with ORF24, ORF31, ORF18, ORF23, and ORF66. Cells (293T) were co-transfected with 2xS-tagged ORF34 and 6xMyc-ORF24 (**a**), 3xFLAG-ORF31 (**b**), 3xFLAG-ORF18 (**c**), 3xFLAG-ORF66 (**d**), 3xFLAG-ORF30 (**e**), or 6xMyc-ORF23 (**f**), upper panel) expression plasmids. Cell lysates were subjected to pull-down assays using S-protein-immobilized beads, which captures S-tagged ORF34. The precipitates were immunoblotted with anti-Myc or FLAG antibody to detect their interactions. (**e**) The pull-down sample was precipitated from cell extracts co-transfected with 2xS-ORF34 and 3xFLAG-ORF31, was applied in the left lane as the positive control. (**f**), lower panel) The pull-down sample was precipitated from cell extracts co-transfected with 2xS-ORF23 and 6xMyc-ORF34. (**a**–**f**) Original images of blotting data are shown in Supplementary Figs S3–S8. (**g**) Schematic depiction of the interactions between KSHV ORF34 and KSHV-PIC component candidates.

To reveal the physical interaction between ORF34 and KSHV transcription-associated factors predicted to be KSHV PIC components (i.e., ORF18, ORF24, ORF30, ORF31, and ORF66), an immunoprecipitation (pull-down) assay was performed. 293 T cells were co-transfected with 2xS-ORF34 and plasmids encoding KSHV-PIC components, and cell lysates were subjected to pull-down assays. As a result, 6xMyc-ORF24 (Fig. 4a), 3xFLAG-ORF31 (Fig. 4b), 3xFLAG-ORF18 (Fig. 4c), and 3xFLAG-ORF66 (Fig. 4d) were found to interact with 2xS-ORF34. However, interaction between 2xS-ORF34 and 3xFLAG-ORF30 was not observed (Fig. 4e). Furthermore, we focused on the KSHV-encoded ORF23 gene as a component of KSHV-PIC because it is highly conserved among beta- and gamma-herpesviruses, and ORF23 protein is known to interact with protein phosphatase 1 (PP1A)^22^, which directly binds to RNAPII^30^. As shown in Fig. 4f, ORF34 was clearly bound to ORF23 protein, indicating that KSHV-PIC contains ORF23 protein in addition to ORF24, ORF31, ORF18, and ORF66 protein as components. A model for the physical interaction between ORF34 and its binding partners are depicted in Fig. 4g.

**Figure 5:**
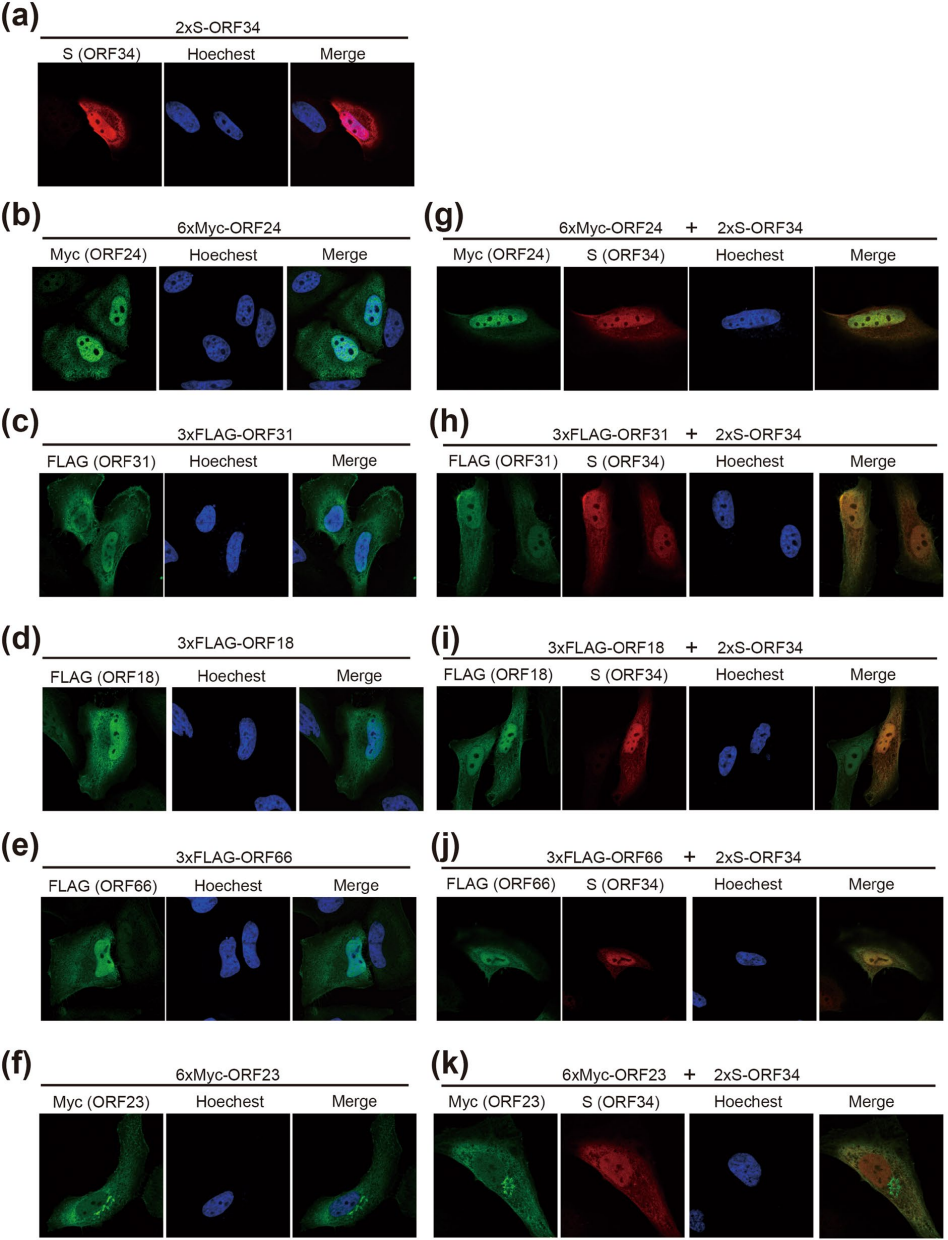
ORF34 colocalizes with ORF24, ORF31, ORF18, ORF23, or ORF66. HeLa cells were also transfected with 2xS-tagged ORF34 (**a**), 6xMyc-ORF24 (**b**), 3xFLAG-ORF31 (**c**), 3xFLAG-ORF18 (**d**), 3xFLAG-ORF66 (**e**), or 6xMyc-ORF23 plasmids (**f**). 2xS-tagged ORF34 plasmid was transfected alone or cotransfected with 6xMyc-ORF24 (**g**), 3xFLAG-ORF31 (**h**), 3xFLAG-ORF18 (**i**), 3xFLAG-ORF66 (**j**), or 6xMyc-ORF23 plasmid (**k**) into HeLa cells, which were then subjected to IFA. Immunofluorescent images were obtained with an inverted confocal microscope. DNA were visualized with Hoechest 33342 staining, and 2xS-tagged ORF34 and the other ORFs (3xFLAG- and 6xMyc-) represented with red and green color, respectively. “Merge” indicates overlaid images of ORF34 (red) and another ORF (green).

To gain insight into the complex formation of KSHV-PIC bearing ORF34 as a central core component, we investigated the colocalization of ORF34 with the other ORFs as well as the interaction of ORF34 with the viral promotor of KSHV lytic genes. First, we analyzed the localization of exogenously transfected ORF34, ORF24, ORF31, ORF18, ORF66, or ORF23 by immunoflourescence analysis (IFA). As a result, ORF34, ORF24, ORF31, ORF18, and ORF66 mainly localized in nuclei and partially in cytosol (Fig. 5a–e), whereas ORF23 localized in nuclei, cytosol, and ER-(or Golgi-) like organelles (Fig. 5f). Furthermore, ORF24, ORF31, ORF18, and ORF66 predominantly colocalized with ORF34 in nuclei (Fig. 5g–j), whereas only a small portion of nuclear-localized ORF23 colocalized with ORF34 in nuclei (Fig. 5k). These data provide further evidence for *in vivo* interactions of ORF34 with the other components of KSHV-PIC. Furthermore, we evaluated whether ORF34 could bind with the viral promoter of L gene (K8.1) or E genes (ORF46/47). The iVero-ΔORF34 cells stably expressing 3xFLAG-ORF34 were treated with NaB and Dox to induce the lytic state, and cells were subjected to a ChIP assay using anti-FLAG antibody. As a result, immunoprecipitates containing ORF34 bound to the promoters of K8.1 (L gene), but failed to bind to the those of ORF46/47 (E gene) (Supplemental Fig. S9). These data indicate that protein complexes containing ORF34 might interact with the L gene promoter through an ORF34-specific interaction with ORF24.

### The central region of ORF34 confers binding of ORF18, ORF31, ORF23, and ORF66, while the C-terminal region confers binding of ORF24 protein and virus production

**Figure 6:**
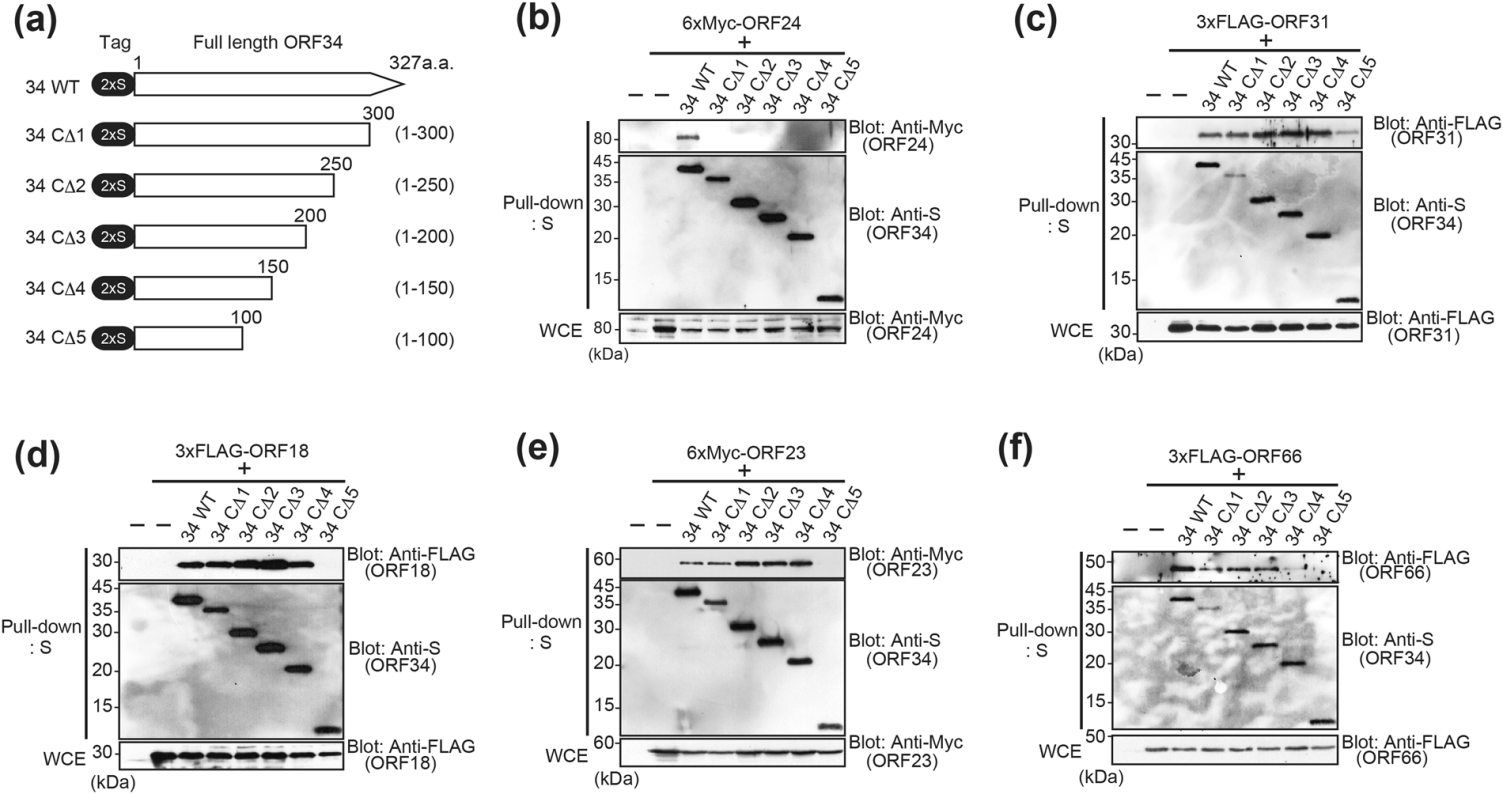
The central region of ORF34 is required for interactions with ORF18, ORF31, ORF23, and ORF66, while the C-terminal domain is required to interact with ORF24. (**a**) Schematic representation of 2xS-tagged ORF34 deletion mutants used in the mapping experiments. The end amino acids are numbered. (**b**–**f**) 293T cells were co-transfected with expression plasmids of 2xS-ORF34 mutant and 6xMyc-ORF24 (**b**), 3xFLAG-ORF31 (**c**), 3xFLAG-ORF18 (**d**), or 3xFLAG-ORF66 (**f**). Transfected cells were lysed, and cell lysates were subjected to pull-down assays using S-protein-immobilized beads that capture the 2xS-ORF34. Obtained precipitates including 2xS-ORF34 deletion mutants were probed with indicated antibodies to detect interaction. (**b**–**f**) Original images of blotting data are shown in Supplementary Figs S10–S14.

The responsible regions of ORF34 for interaction with ORF24, ORF31, ORF18, ORF23, and ORF66 protein were identified. A schematic of 2xS-tagged ORF34 deletion mutants is depicted in (Fig. 6a). Using these 2xS-ORF34 deletion mutants, the interaction domains of ORF34 with 6xMyc-ORF24 (Fig. 6b), 3xFLAG-ORF31 (Fig. 6c), 3xFLAG-ORF18 (Fig. 6d), 6xMyc-ORF23 (Fig. 6e), or 3xFLAG-ORF66 (Fig. 6f) were mapped by pull-down assays. Data show that the central region of ORF34 interacts with ORF18, ORF31, ORF23, and ORF66 protein, whereas C-terminal region of ORF34 interacts with ORF24 protein. Since ORF34 binds with predicted KSHV-PIC components through its central or C-terminal region, full length ORF34 appears to be indispensable for assembly of KSHV-PIC.

**Figure 7:**
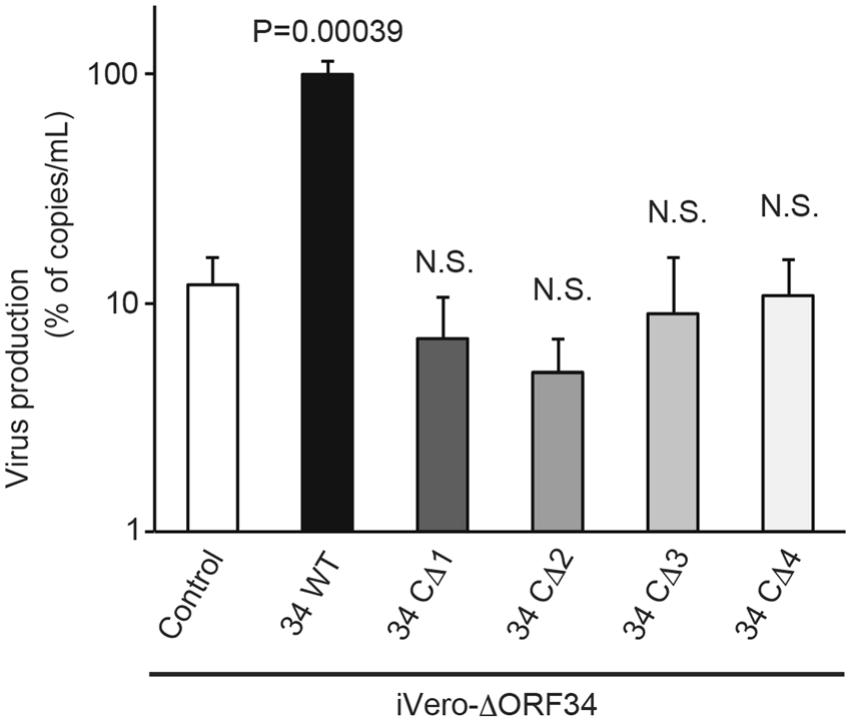
The interaction of the ORF34 C-terminal region and ORF24 is essential for virus production. The iVero-ΔORF34 cells were transfected with control, ORF34 or a ORF34-deletion mutant plasmid. After 2 days, transfected cells were stimulated with NaB and Dox for 3 days. Progeny KSHV was purified from harvested culture supernatant, and the KSHV genome was quantified by real-time PCR. Viral productivities were assessed using three independent samples, and error bars indicate standard deviations. Statistical significance between each group and the control was analyzed by two-tailed Student’s *t*-test (N.S.: not significant, P > 0.05).

KSHV ORF24 interacts with TATT sequences in the KSHV L gene promoter, RNAPII^22^, and ORF34^23^ that bound to ORF18, 31, 23, and 66 (Figs 4 and 6). In other words, the binding between ORF34-ORF24 may serve as a core scaffold for the assembly of the viral transcription initiation complex (predicted vPIC) with RNAPII and the L gene promoter^23^. Because mapping data shows that ORF34 interacts with ORF24 protein via its C-terminal region, we therefore investigated whether this interaction is important for virus production by *trans*-complementation assay using exogenous ORF34 mutants lacking variable lengths of the C-terminal region. ORF34 mutant plasmids were transfected into iVero-ΔORF34, and recovery of virus production was measured. Wild type ORF34 expression significantly increased viral production in iVero-ΔORF34 cells, while recovery of virus production by all ORF34 deletion mutants was not detected (Fig. 7). ORF34 C-terminal deletion mutants, which lose the ability to interact with ORF24, failed to rescue viral production in iVero- ΔORF34 cells. These data indicate that the central region of ORF34 recruits transcription associated factors, and the C-terminal region plays a crucial role in the recruitment of this complex to ORF24, which binds to the L gene promoter of viral DNA. Thus, full length ORF34 is essential for L gene expression and viral production.

## Discussion

Our study reveals the importance of ORF34 for KSHV gene expression through the formation of the predicted KSHV-PIC. In particular, we found that: (i) ORF34-deficient KSHV failed to produce progeny virus and express L genes; (ii) ORF23 is a novel vPIC component; (iii) the central region of ORF34 interacts with transcription associated factors (ORF18, 31, 23, and 66) of the predicted KSHV-PIC; and (iv) the C-terminal region of ORF34 interacts with the transcription-complex core-component ORF24. We also revealed that viral production of iVero-ΔORF34 cells are rescued by exogenous full length ORF34 expression but not by the expression of ORF34 C-terminal deletion mutants (Fig. 7). These findings indicate that full length ORF34 functions as a critical component for the assembly of the KSHV-PIC transcription complex. On the other hand, ORF34 C-terminal deletion mutants resulted in lower viral yields (less than 10%), compared with ORF34 wild type. We speculate that cellular basal transcription factors, such as TFIIB, TAFs and TBP, may be able to assemble into KSHV-PIC independent of ORF34 in L gene expression and viral production to a certain extent, but that this activity is highly attenuated without full length ORF34. In gamma-herpesvirus EBV and MHV68 (murine gamma herpes-68), EBV BGLF3 and MHV68 ORF34 are KSHV ORF34 orthologs. The siRNA-knockdown of EBV-BGLF3^31^ and MHV68-ORF34 deficient virus^27^ caused a reduction of their L gene expression. In beta-herpesvirus CMV (Cytomegalovirus), the inhibition of L gene expression was conferred by the loss of CMV-UL95, a KSHV-ORF34 ortholog^32^. This evidence suggests that the function and features of beta- and gamma-herpesvirus ORF34 orthologs seem to be conserved among CMV, EBV, MHV, and KSHV.

ORF23 is a highly conserved gene among beta- and gamma-herpesviruses, suggesting an important role for virus replication but the function of KSHV ORF23 is currently unknown. However, MHV68 ORF23, a KSHV ORF23 ortholog, was reported as a non-essential gene for virus production^33^. KSHV ORF23 has been reported to interact with the protein phosphatase 1-alpha catalytic subunit (PP-1A)^22^ which belongs to the serine/threonine-protein phosphatase family. It is well known that the PP-1 family regulates a number of proteins through dephosphorylation. Interestingly, RNAPII is dephosphorylated by PP-1A^30^. The specific dephosphorylation of RNAPII is necessary for RNAPII recruitment to pre-initiation complexes for transcription^34^. Therefore, we speculate that ORF23 may be recruited to the KSHV-PIC through an interaction with ORF34, leading to ORF23 binding with PP-1A, and subsequent dephosphorylation of RNAPII. Further investigation is necessary to elucidate the role of ORF23 during the KSHV lifecycle.

In this study we revealed that ORF24, ORF31, ORF18, ORF66, and ORF23 interact with ORF34 by pull-down assays (Fig. 4). The interaction between ORF34 and ORF30 was not detected (Fig. 4e), which is in agreement with previous reports^23, 24^. ORF18 binds both ORF30 and ORF34, and serves as a bridge between the two molecules^23^. Similarly, ORF34 bridges ORF24 and ORF31 together^24^, with ORF24 binding to RNAPII^22^, ORF34^23^, and TATT sequences in K8.1 and ORF57 promoters^23, 24^. Our findings are in line with a previous result that showed ORF34 associated with ORF18, ORF31, and ORF66 by a split luciferase assay^23^. Taken together, KSHV-PIC appears to consist of KSHV ORFs 18, 23, 24, 30, 31, 34, and 66, as well as host RNAPII. Our results suggest that KSHV ORF34 serves as a hub for the recruitment of a transcription complex (ORF18, 23, 30, 31, and 66) to ORF24 on TATT sequences in the L gene promoter region.

ORF34 deficiency significantly decreased virus production (Fig. 2b) and L gene expression (Fig. 3) in iVero-ΔORF34 cells but did not affect KSHV genomic DNA replication (Fig. 2c) nor the expression of latent and E genes (Fig. 3). These results are in agreement with previous reports that the ablation of other ORF34-interacting ORFs (ORF24, ORF31, ORF18, or ORF30) had no effect on viral DNA replication^21, 22, 24^. Furthermore, in EBV and KSHV, viral transcription associated factors related to vPIC, contribute to the transcription of the L genes in a manner that is delineated from viral DNA replication and IE and E gene expression^16^.

Our findings shed light on the importance of ORF34 and the composition of the L gene viral transcription machinery during KSHV infection, contributing to our understanding of beta- and gamma-herpesviruses. Furthermore, these findings suggest that inhibitory molecules, which can inhibit the function of ORF34 or the interaction between ORF24 and ORF34, may present a novel strategy for the treatment of KSHV infection in the future.

## Methods

### Plasmids

To construct pENTR4-KSHV RTA/ORF50, KSHV RTA/ORF50 fragments were obtained by PCR from KSHV genome harboring PEL cells (BC3) and inserted into pENTR4 no ccdB (686-1), a gift from Dr. Eric Campeau (Addgene plasmid # 17424)^35^. To construct pCW57.1-KSHV RTA/ORF50, the KSHV RTA/ORF50 fragment was transferred from pENTR4-KSHV RTA/ORF50 to a Tet-ON lentiviral vector pCW57.1, a kind gift from Dr. David Root (Addgene plasmid # 41393), by the GATEWAY cloning (Invitrogen, CA, USA). KSHV ORF18, 23, 24, 30, 31, 34, 66 coding fragments were obtained by PCR from BAC16 using primer sets noted in Supplementary Table S1, and were cloned into pCI-neo-2xS, pCI-neo-6xMyc, and pCI-neo-3xFLAG vectors, which had been generated by inserting oligonucleotides encoding two repeats of an S-tag peptide, six repeats of a Myc-tag sequence, and three repeats of a FLAG-tag sequence, respectively, into the pCI-neo mammalian expression vector (Promega, WI, USA).

### Mutagenesis of KSHV BAC16

KSHV BAC16 was a kind gift from Jae U. Jung and mutagenesis of KSHV BAC16 was performed according to their protocols^25, 26^. The primers for mutagenesis sequences are noted in Supplementary Table S1. Insertion and deletion of kanamycin resistance cassettes (Kan^R^) in each mutant were analyzed by digestion of EcoRI and agarose-gel electrophoresis. Mutated sites of each BAC clone were confirmed by Sanger sequencing.

### Establishment of tetracycline/doxycicline-inducible recombinant KSHV-expressing Vero cells

The lentivirus vector was produced by transfection of pCMV-VSV-G-RSV-Rev, pCAG-HIVgp, and pCW57.1-KSHV RTA/ORF50 into 293T cells. Packaging plasmids (pCMV-VSV-G-RSV-Rev, pCAG-HIVgp) were a kind gift from Dr. Hiroyuki Miyoshi (RIKEN, JAPAN). To establish RTA/ORF50 inducible Vero cells (iVero cells), Vero cells were transfected with the lentivirus vector as described previously^36, 37^. For selection and maintenance, cells were cultured in a growth medium containing 2.5 μg/mL of puromycin (InvivoGen, CA, USA). KSHV BAC16 wild-type (WT-BAC16) and mutants (ΔORF34-BAC16 and Revertant-BAC16) were transfected to iVero cells by a calcium phosphate method^38^. Transfected cells were selected under 1000 μg/mL of hygromycin B (Wako, Osaka, Japan) and 2.5 μg/mL of puromycin to establish doxycycline-inducible recombinant KSHV producing cell lines (iVero-WT, iVero-ΔORF34, iVero-Revertant).

### Production and infection of recombinant KSHV

Infection assays of recombinant KSHV were performed as described previously^26^. Briefly, iVero cell lines harboring KSHV BAC (iVero-WT, iVero-ΔORF34 or iVero-Revertant) were treated with 8 μg/mL of doxycycline (Dox) (LKT Laboratories, MN, USA) and 1.5 mM sodium butyrate (NaB) (Tokyo Chemical Industry, Tokyo, Japan) for 96 hours. Culture supernatants were filtered and ultra-centrifuged, and precipitates containing virus were suspended with culture media. Concentrated viruses were inoculated onto Vero or 293T cells in the presence of 8 μg/mL of polybrene (Sigma-Aldrich, MO, USA). After 48 hours, infectivity (GFP positive cells) was analyzed by FACS Calibur (Beckton Dickinson, CA, USA). NaB binds HDACs and induces hyperacetylation of histones, resulting in transcriptional activation. Dual treatment of both Dox and NaB was used in this study because it induces RTA expression and the lytic cycle more effectively.

### Measurement of virus production and viral DNA replication

For quantification of virus production, KSHV virions in culture supernatant were quantified as previously described^39, 40^. Briefly, iVero cells (iVero-WT, iVero-ΔORF34 or iVero-Revertant) were treated with 8 μg/mL of doxycycline and 1.5 mM of NaB for 48 hours to induce to lytic replication and production of recombinant KSHV, and culture supernatants were harvested. Culture supernatants (300 μL) were treated with DNase I (NEB, MA, USA) to obtain only enveloped and encapsidated viral genomes. Viral DNA were purified and extracted from 200 μL of DNase I-treated culture supernatant using the QIAamp DNA blood mini kit (QIAGEN, CA, USA). To quantify viral DNA copies, SYBR green real-time PCR was performed using KSHV-encoded ORF11 specific primers, listed in Supplementary Table S1. For measurement of KSHV genome replication, iVero cells were treated with 8 μg/mL of doxycycline and 1.5 mM of NaB for 48 hours to induce lytic replication, and harvested. Total cellular DNA containing the cellular KSHV genome were purified and extracted from washed cells using the QIAamp DNA blood mini kit (QIAGEN). Cellular KSHV genome copies were determined by SYBR green real-time PCR and normalized to total DNA concentration.

### Recovery of exogenous gene in BAC harboring cells

The cells were transfected with pCI-neo-2xS as a control plasmid, and 2xS-taggged ORF34 full length and deletion mutant plasmids using Screenfect A plus (Wako, Tokyo, JAPAN) according to the manufacturer’s instructions. After two days, transfected cells were stimulated with NaB 0.5 mM/Dox 8 μg/mL containing medium. After three days of stimulation, viral supernatant was harvested and KSHV genome was evaluated by real-time PCR.

### RT real-time PCR (RT-qPCR)

mRNA was extracted from each iVero cells treated with Dox and NaB using RNAiso Plus (TAKARA, Osaka, Japan). cDNA was synthesized by Revetra Ace qPCR kit (TOYOBO, Osaka, Japan) and subjected to SYBR green real-time PCR. The sequences of RT-qPCR primer sets were noted in Supplementary Table S1. Relative mRNA expression levels were determined by GAPDH expression and ΔΔCt methods.

### Cell culture, western blot, pull-down assay and antibodies

293T and Vero cells were cultured in DMEM supplemented with 10% fetal calf serum. Western blots were performed as described previously^39, 41^. For pull-down assays, transfected 293T cells were lysed by HNTG buffer (20 mM HEPES (pH 7.9), 0.18 M NaCl, 0.1% NP-40, 0.1 mM EDTA, 10% Glycerol)^16^ with protease inhibitors and sonicated. The cell extracts were subjected to affinity purification using S-protein-immobilized beads (Novagen, MA, USA), and purified proteins (containing 2xS-tagged ORF34 or mutants) were subjected to western blotting. Anti-Myc (9E10) (Calbiochem, MA, USA), Anti-S-probe (K-14) (Santa-Cruz, CA, USA), Anti-FLAG (M2) (Sigma-aldrich) were used as the primary antibodies. HRP linked anti-mouse IgG antibody (GE healthcare UK Ltd., Buckinghamshire, UK) or HRP linked anti-rabbit IgG antibody (GE healthcare UK Ltd.) was used as the secondary antibody.

### Immunofluorescence analysis (IFA)

HeLa cells seeded on glass slides were co-transfected with 2xS-tagged ORF34 and 3xFLAG- (or 6xMyc-) tagged ORFs and fixed with 4% *p*-formaldehyde at room temperature for 20 min, and then methanol at −20 °C for 1 hour. Cells were incubated with Anti-Myc, Anti-FLAG, Anti-S-probe antibodies. After washing, the cells were further incubated with Alexa fluor 488 conjugated anti-mouse IgG, Alexa fluor 594 conjugated anti-rabbit IgG and Hoechst 33342. Immunofluorescent images were obtained with an inverted confocal microscope (Nikon A1R+; Nikon, Tokyo, Japan).

### Chromatin Immuno-precipitation (ChIP)-qPCR assay

ChIP assays were performed as described previously^42^. Briefly, iVero-ΔORF34/3xFLAG-ORF34 cells were treated with or without 8 μg/mL of Dox and 1.5 mM NaB for 72 hours. Soluble chromatin were prepared from formaldehyde-fixed cells by sonication and then subjected to immunoprecipitation with an anti-FLAG (M2) monoclonal antibody (Sigma-Aldrich) or a mouse control IgG (Santa-Cruz). Immunoprecipitates containing chromatin and viral DNA were subjected to SYBR green real-time PCR for measuring the levels of promoter DNA of ORF46/47 (E gene) or K8.1 (L gene). The levels of immunoprecipitated viral DNA were normalized to total input DNA. The sequences of qPCR primer sets for each ORF transcriptional start site of are noted in Supplementary Table S1.

### Statistics

The two-tailed student’s *t*-test was used to indicate the differences between the groups. *P* values are shown in each figure.

## Electronic supplementary material

Supplementary Figure and table
