# Supplementary material for: Kaposi’s sarcoma-associated herpesvirus ORF34 is essential for late gene expression and virus production
Source: Sci Rep. 2017 Mar 23;7:329. doi: 10.1038/s41598-017-00401-7 (PMC5428543; doi:10.1038/s41598-017-00401-7)
Supplement: Supplementary file 1 — Supplementary Figure and table [file 41598_2017_401_MOESM1_ESM.pdf]

Supplementary information

Kaposi's sarcoma-associated herpesvirus ORF34 is essential for late gene expression and virus production

Mayu Nishimura, Tadashi Watanabe, Shota Yagi, Takahiro Yamanaka and Masahiro Fujimuro*

Department of Cell Biology, Kyoto Pharmaceutical University, Misasagi-Shichono-cho 1, Yamashina-ku, Kyoto 607-8412, Japan.

*: Address correspondence to Masahiro Fujimuro, PhD. Department of Cell Biology, Kyoto Pharmaceutical University, Misasagi-Shichono-cho 1, Yamashina-ku, Kyoto 607-8412, Japan; Tel: +81-75-595-4717
E-mail: fuji2@mb.kyoto-phu.ac.jp

Supplementary Table S1: Primers for construction of BACmid/ expression plasmid and real-time PCR/ RT-qPCR

Primer name	Primer sequences (5' -> 3')
**[BAC mutagenesis]**	**a*
BACmut-ORF34-3stop-F	aactcccctgaacagcttcctcttggtttccaacgtgatgTAGTTAGATAGTcccgaggtccagccaatctgTAGGGATAACAGGGTAATCGATTT
BACmut-ORF34-3stop-R	gcaaggccggccggccactgcagattggctggacctcgggACTATCTAACTAcatcacgttggaaaccaagaGCCAGTGTTACAACCAATTAACC
BACmut-ORF34-Rev-F	aactcccctgaacagcttcctcttggtttccaacgtgatgcccgaggtccagccaatctgTAGGGATAACAGGGTAATCGATTT
BACmut-ORF34-Rev-R	gcaaggccggccggccactgcagattggctggacctcgggcatcacgttggaaaccaagaGCCAGTGTTACAACCAATTAACC
**[Cloning expression plasmid]**	**b*
cloning_ORF34-F	acatgaattcATGTTTGCTTTGAGCTCGC
cloning_ORF34-R	ctaagtcgacTTAGAGTTGGTTGAGTCCATTCTCC
cloning_ORF34D1-R	ttaagtcgacTTAAGTTGGACACCTTTGGGTC
cloning_ORF34D2-R	ttaagtcgacTTAAGTGTCGGAAGCATCTATG
cloning_ORF34D3-R	ttaagtcgacTTAAAGATCCCGTTTGGGAGC
cloning_ORF34D4-R	ttaagtcgacTTAAAGACCCTTCCAAGTTCCG
cloning_ORF34D5-R	taagtcgacTTAGTCCAGTGCGATTTCCTCAC
cloning_ORF18-F	catgaattcATGCTCGGAAAATACGTGTGTG
cloning_ORF18-R	taaacgcgtTTAAACCGCGTTGTTGTTAAAC
cloning_ORF23-F	cattctagaATGTTACGAGTTCCGGACGTGAAGGC
cloning_ORF23-R	taagcggccgcTTAGACGGTCAATAAAGCG
cloning_ORF24-F	atacgcgtATGGCAGCGCTCGAGG
cloning_ORF24-R	taatctagaTTAGACCAGCGGACGGACGC
cloning_ORF30-F	catgaattcATGGGTGAGCCAGTGGATCCTG
cloning_ORF30-R	tgaacgcgtTCATTTCGCACCGGTGTC
cloning_ORF31-F	catgaattcATGTCACAAAACAGAAAGACTCTGC
cloning_ORF31-R	tagacgcgtCTACGTATCTTTCGTTGATAGC
cloning_ORF66-F	cattctagaATGGCCCTGGATCAGCGCTGGGATC
cloning_ORF66-R	gagcggccgcTCAGGAGGAACACTTCCC
**[qPCR/ RT-qPCR]**	
real-time_PCR_KSHV ORF11-F	TTGACAACACGCACCGCAAG
real-time_PCR_KSHV ORF11-R	AAAAATCAGCACGCTCGAGGAG
RT-qPCR_ORF72/vFLIP-F	CTGGACTTTTGGCACCACGAG
RT-qPCR_ORF72/vFLIP-R	AGCGCTGATAATAGAGGCGGG
RT-qPCR_ORF16-F	AGATTTCACAGCACCACCGGTA
RT-qPCR_ORF16-R	CCCCAGTTCATGTTTCCATCGC
RT-qPCR_ORF46-F	CACTGCTGCGATCCAGAGGATA
RT-qPCR_ORF46-R	GAACCTGACATTGCGGATCCAC
RT-qPCR_ORF59-F	AAGGGGAAGAAGTCGGTGGATG
RT-qPCR_ORF59-R	AACCAACCCGGGACTTTACACA
RT-qPCR_K8.1-F	TAAACGGGACCAGACTAGCAGC
RT-qPCR_K8.1-R	GTTTTCTGCGACCGGTGATACG
RT-qPCR_ORF26-F	AAGGGGAAGAAGTCGGTGGATG
RT-qPCR_ORF26-R	AACCAACCCGGGACTTTACACA
RT-qPCR_ORF25-F	TACGGTCTCAGGTATGAGCAGA
RT-qPCR_ORF25-R	GTAGATCCTCCGTGGTTACGAG
RT-qPCR_ORF27-F	GACGCATTTACCCGAACTCTAC
RT-qPCR_ORF27-R	TGCACATGACGTGTTAACCATA
RT-qPCR_ORF42-F	TCTCCAGGTGCTTGGTAAAGAT
RT-qPCR_ORF42-R	TAAGCTTCATAAGGCGATAGGC
RT-qPCR_ORF43-F	TCCTGTAAACGTCCCAGAGATT
RT-qPCR_ORF43-R	TGTCTCACCAACCAGATAAACG
RT-qPCR_ORF53-F	CTAAAACTATCCGCGGAACAAG
RT-qPCR_ORF53-R	GCCTCGATACTAGGTCACTGCT
RT-qPCR_ORF55-F	GAGGCAATACAGAAGTGGGTTC
RT-qPCR_ORF55-R	GCTCCAGTCCCTCTTAAACAAA
RT-qPCR_ORF65-F	GTTGTGAGAATGTCTGACGCCG
RT-qPCR_ORF65-R	GGTGGAATTCAAAGGCGGGATC
RT-qPCR_ORF68-F	TTCTGTCTATACGCCCCACACTG
RT-qPCR_ORF68-R	CCTCCTCTTCTGGAAAGTGCAGA
RT-qPCR_ORF52-F	ACAAAAGCAAGTGGACGATGCC
RT-qPCR_ORF52-R	CTCTTCGTCGCCTGTTATTGGC
RT-qPCR_ORF8-F	TGTATCCGCCAAGTTCGTAGGAG
RT-qPCR_ORF8-R	GCTCTTGTGGATGTTTACGGAGC
RT-qPCR_GAPDH-F	CATCAAGAAGGTGGTGAAGCAG
RT-qPCR_GAPDH-R	TGTCGCTGTTGAAGTCAGAGG
**[CHIP qPCR]**	
CHIP-qPCR_ORF46/47-F	AGCCCCCTTCCGTAATATCTG
CHIP-qPCR_ORF46/47-R	TTTTCCGCGGAAGTATGTCG
CHIP-qPCR_K8.1-F	ACTCCCACCATGTTGAAGCTTG
CHIP-qPCR_K8.1-R	GGGATTTCTGTGCGAATCTGTG

**a*: Lowercase indicates homology sequence to KSHV BAC16, underlined uppercase indicates mutagenesis site, and uppercase indicates pEP-KanS sequence

**b*: underlined lowercase indicates mutagenesis site, and uppercase indicates KSHV BAC16 ORF sequences

Supplementary Fig. S1 (original data of Fig. 1b)

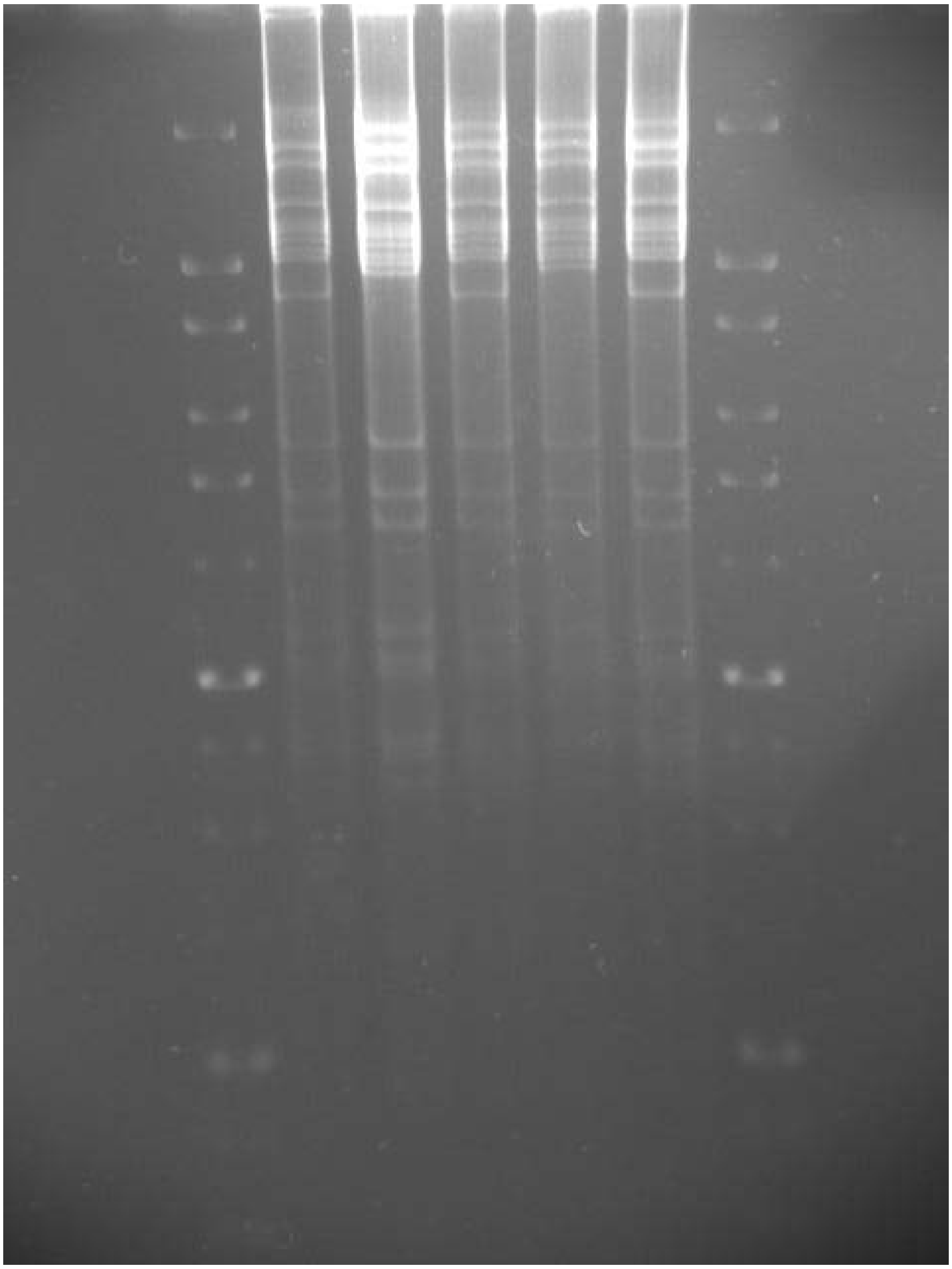

Supplementary Fig. S2

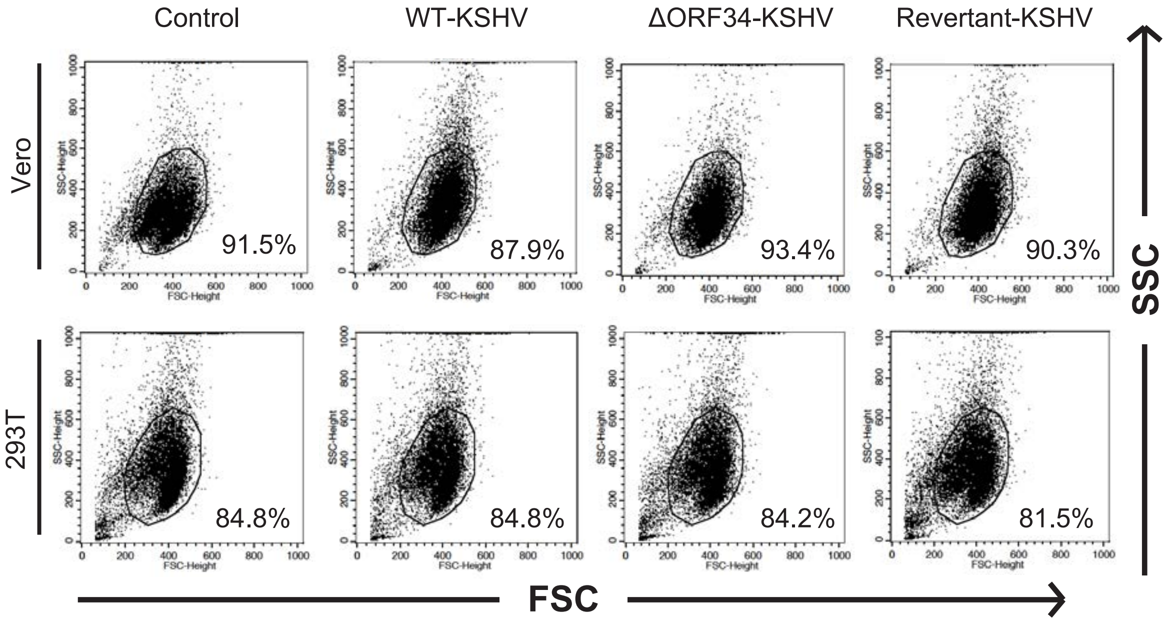

Supplementary Fig. S3 (original data of Figure 4a)

Whole cell extract | Pull-down: S-Agarose

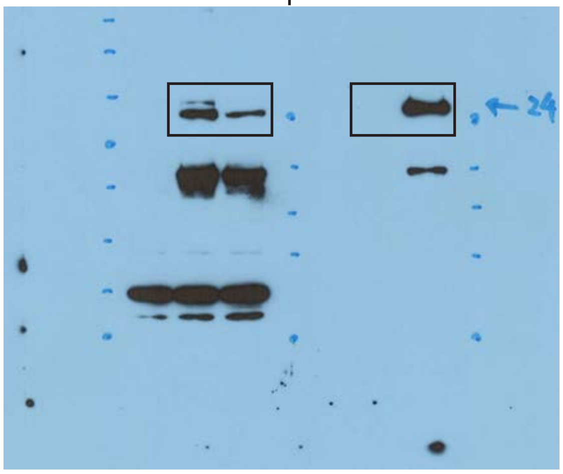

Blot: Myc

Whole cell extract | Pull-down: S-Agarose

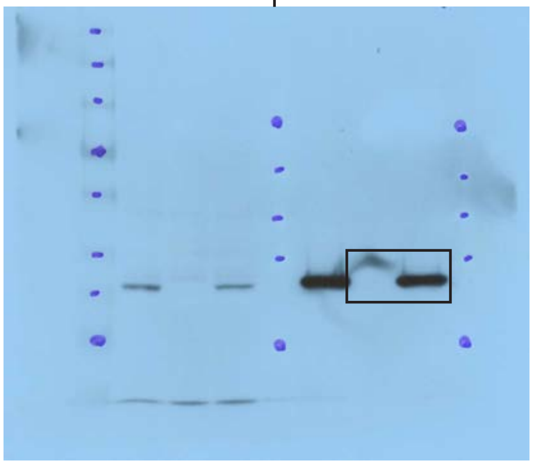

Blot: S

Supplementary Fig. S4 (original data of Figure 4b)

Pull-down: S-Agarose

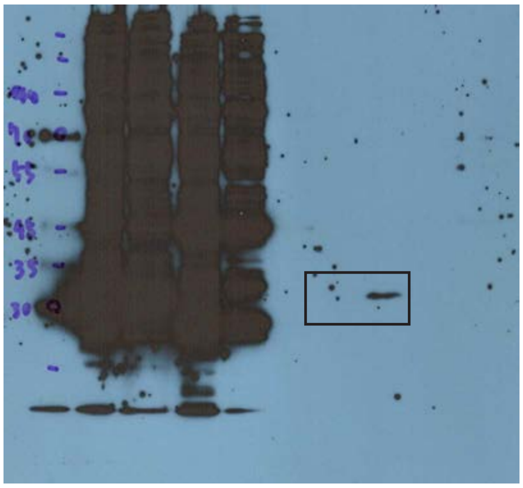

Blot: FLAG

Pull-down: S-Agarose

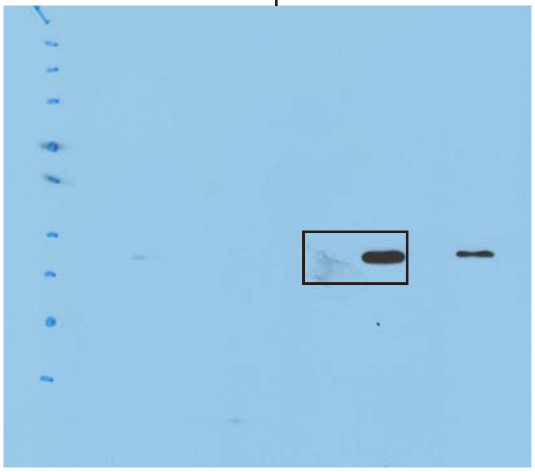

Blot: S

Whole cell extract

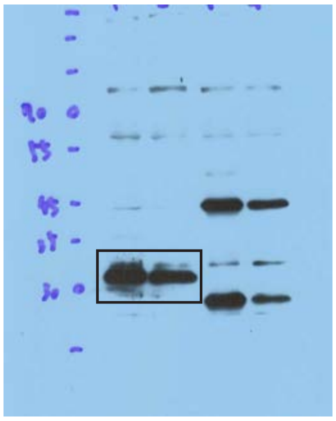

Blot: FLAG

Supplementary Fig. S5 (original data of Figure 4c)

Pull-down: S-Agarose

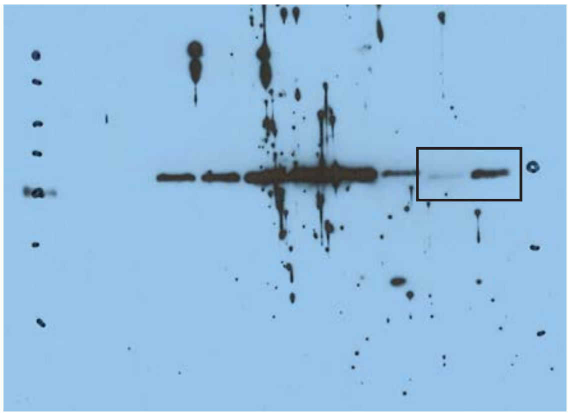

Blot: FLAG

Pull-down: S-Agarose

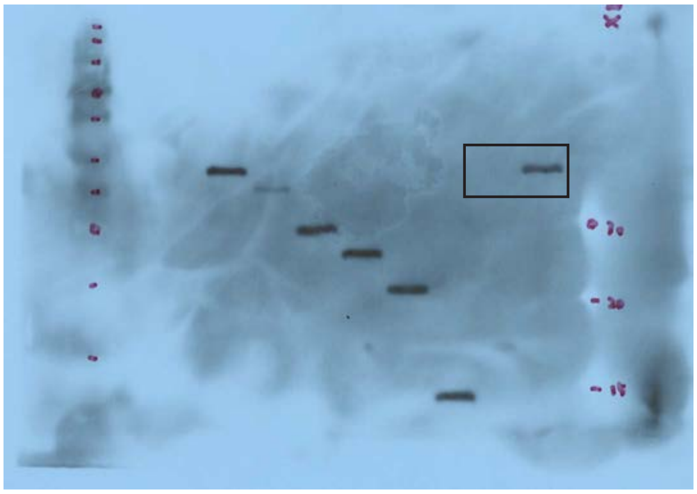

Blot: S

Whole cell extract

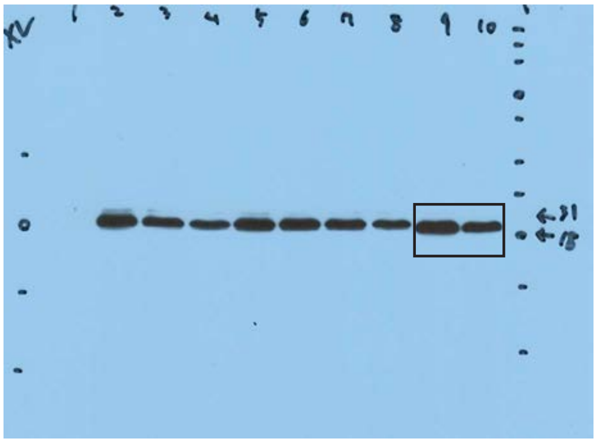

Blot: FLAG

Supplementary Fig. S6 (original data of Figure 4d)

Pull-down: S-Agarose | Whole cell extract

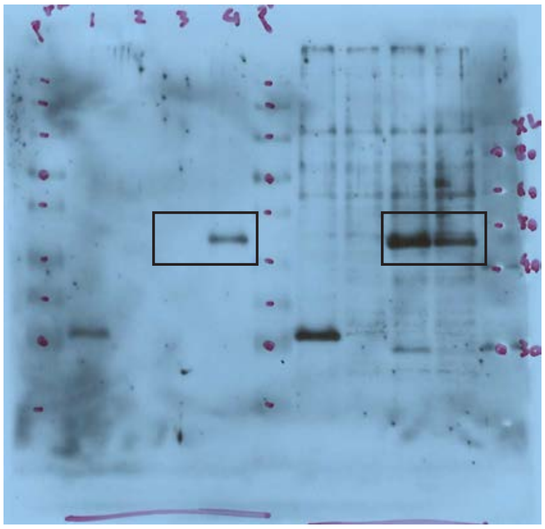

Blot: FLAG

Pull-down: S-Agarose

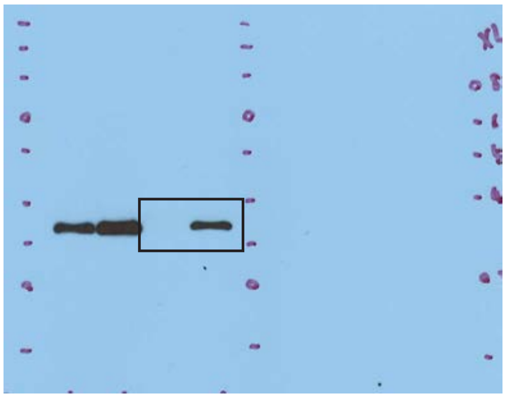

Blot: S

Supplementary Fig. S7 (original data of Figure 4e)

Pull-down: S-Agarose | Whole cell extract

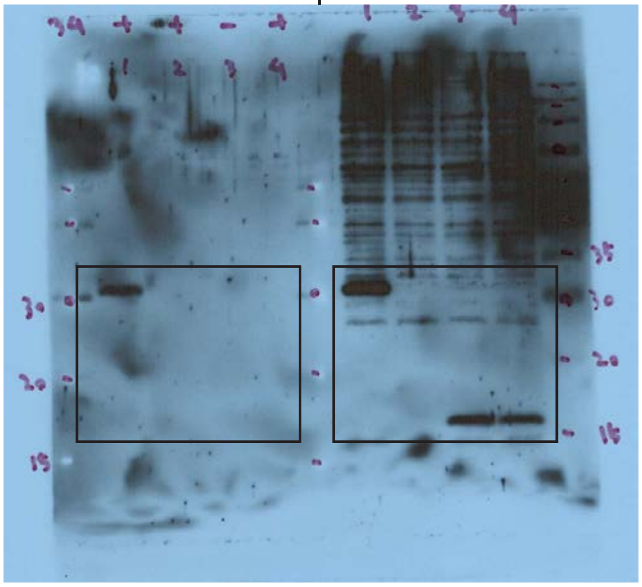

Blot: FLAG

Pull-down: S-Agarose

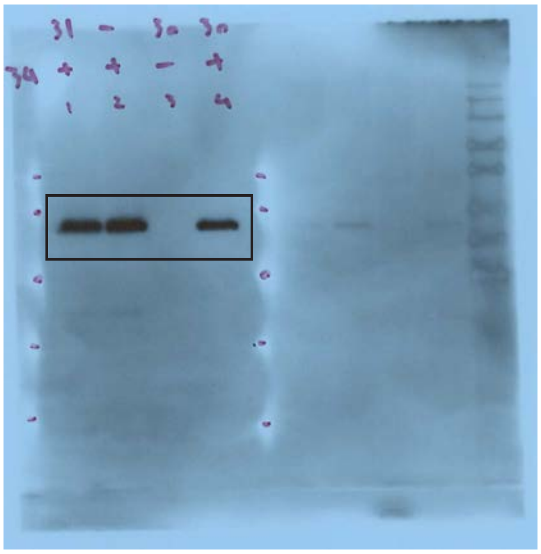

Blot: S

Supplementary Fig. S8 (original data of Figure 4f)

Pull-down: S-Agarose | Whole cell extract

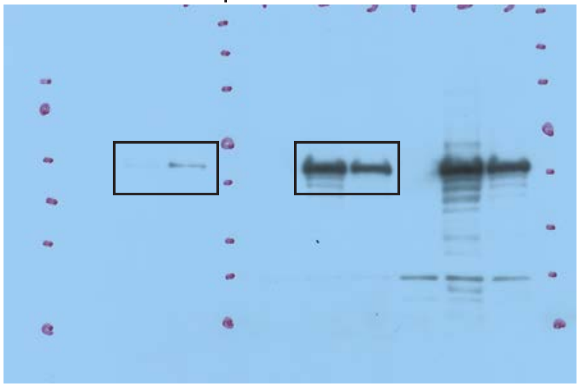

Blot: Myc

Pull-down: S-Agarose

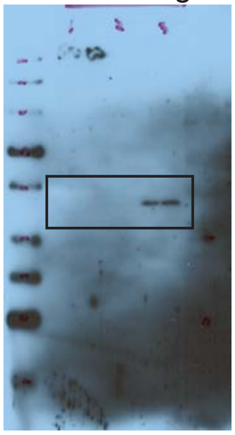

Blot: Myc

Whole cell extract

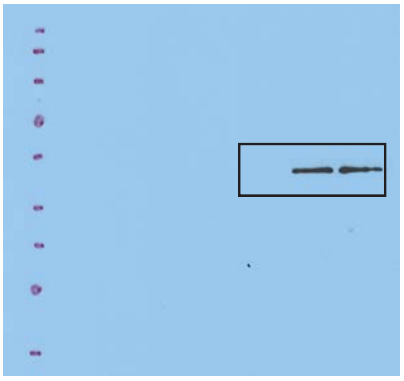

Blot: Myc

Pull-down: S-Agarose

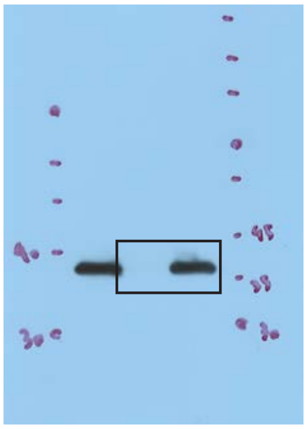

Blot: S

Pull-down: S-Agarose

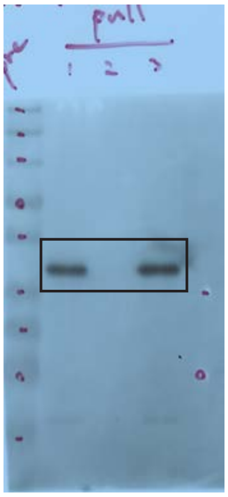

Blot: S

Supplementary Fig. S9

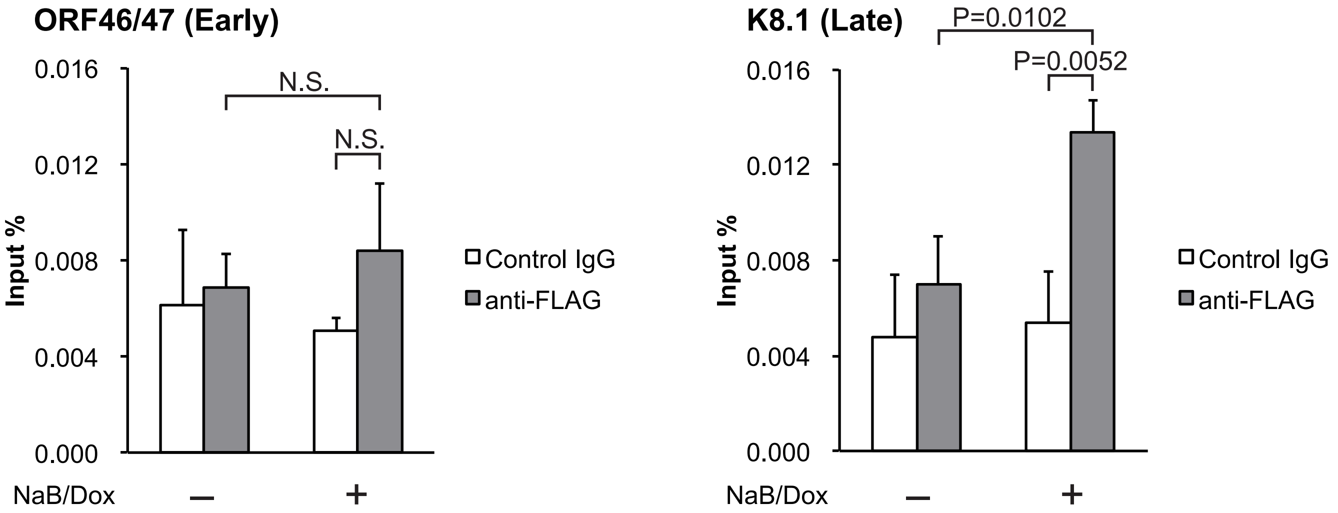

To establish iVero-ΔORF34 cells stably expressing 3xFLAG-ORF34 protein, iVero-ΔORF34 cells were transfected with 3xFLAG-tagged ORF34 plasmid, and then selected and maintained with media including 1.5 mg/mL G418. 3xFLAG-ORF34-expressing iVero-ΔORF34 cells were treated with (or without) Dox and NaB for 72 hours and subjected to ChIP-qPCR. 3xFLAG-tagged ORF34 protein was Immunoprecipitated by anti-FLAG or control antibody, and precipitates including chromatin and viral DNA were subjected to SYBR green real-time PCR for measuring the amount of promoter DNA of ORF46/47 (E gene) or K8.1 (L gene). The levels of immunoprecipitated viral promoter were normalized to total input DNA.

Supplementary Fig. S10 (original data of Figure 5b)

Pull-down: S-Agarose

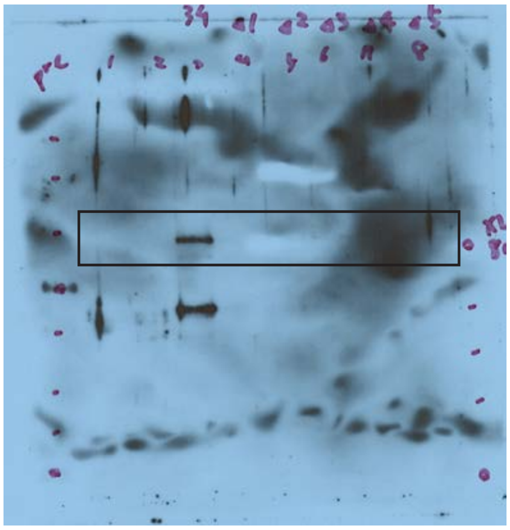

Blot: Myc

Pull-down: S-Agarose

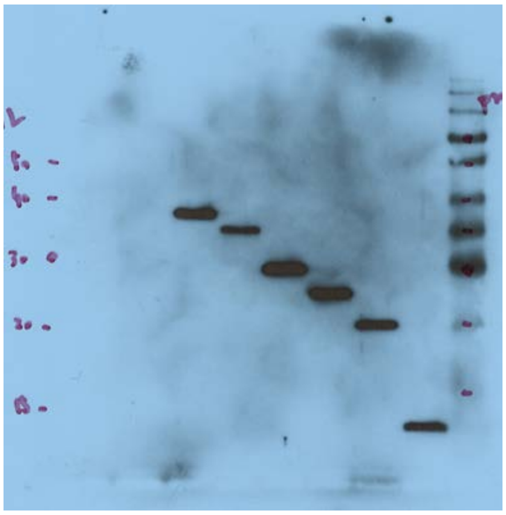

Blot: S

Whole cell extract

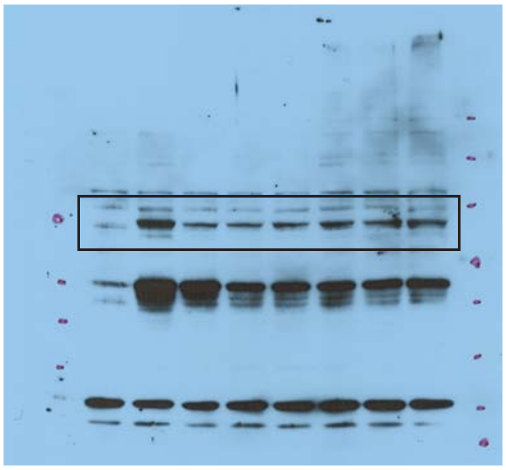

Blot: Myc

Supplementary Fig. S11 (original data of Figure 5c)

Pull-down: S-Agarose

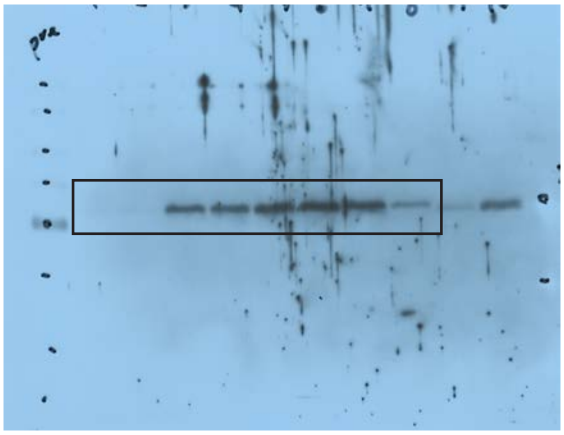

Blot: FLAG

Pull-down: S-Agarose

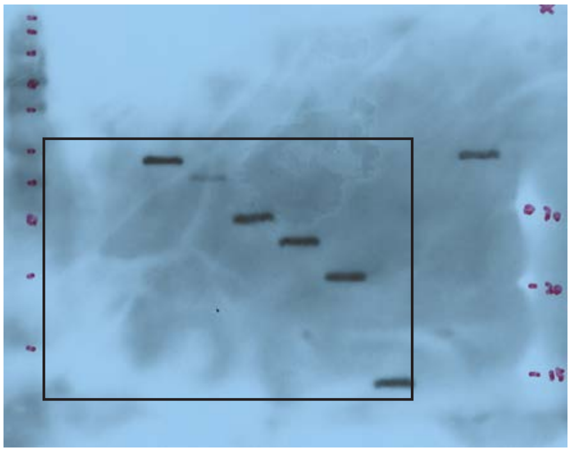

Blot: S

Whole cell extract

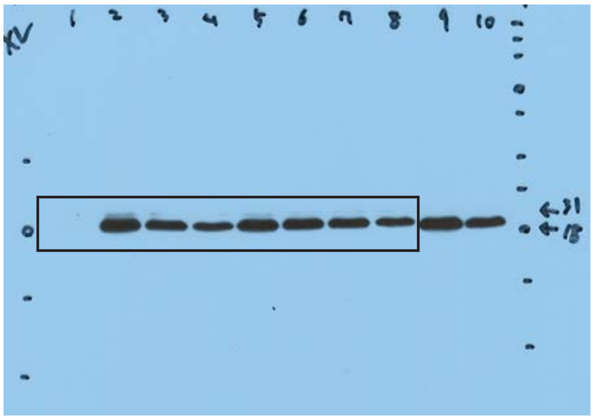

Blot: FLAG

Supplementary Fig. S12 (original data of Figure 5d)

Pull-down: S-Agarose

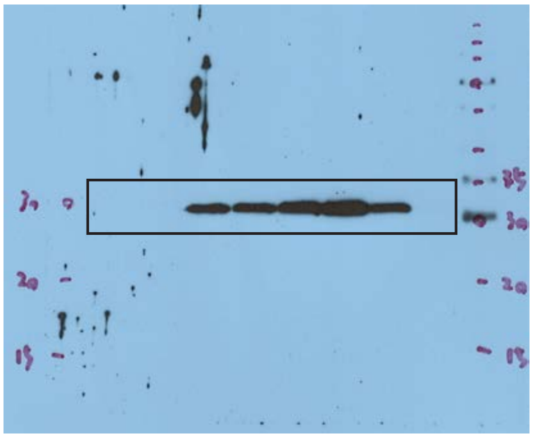

Blot: FLAG

Pull-down: S-Agarose

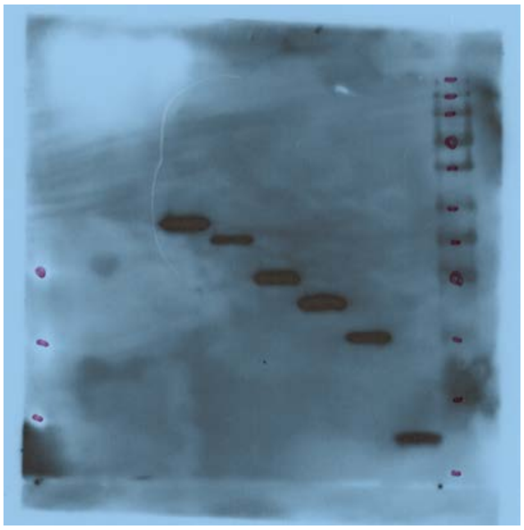

Blot: S

Whole cell extract

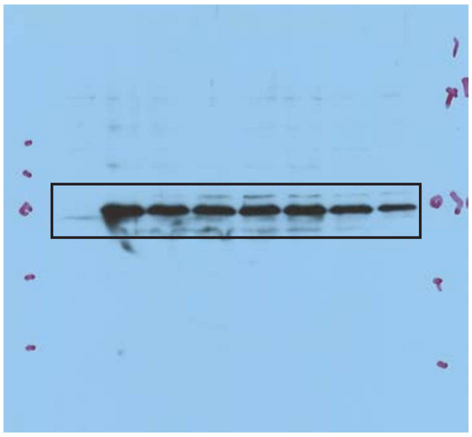

Blot: FLAG

Supplementary Fig. S13 (original data of Figure 5e)

Pull-down: S-Agarose

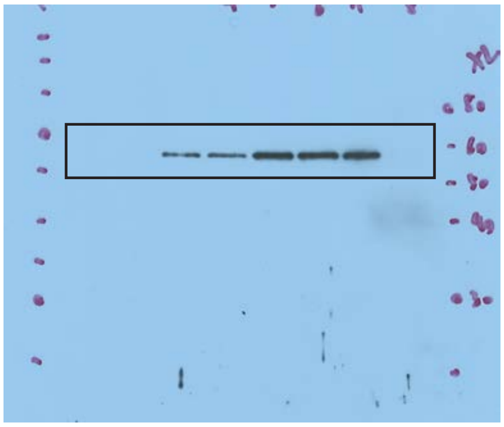

Blot: Myc

Pull-down: S-Agarose

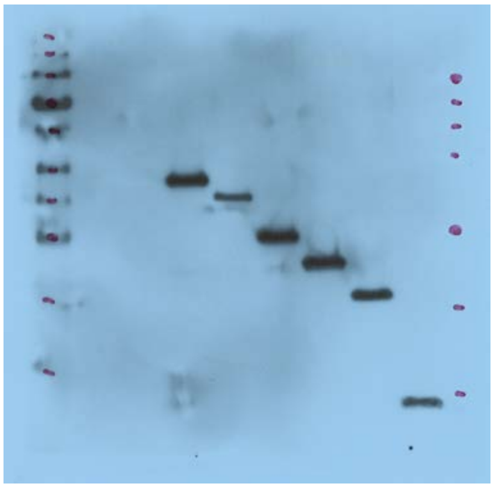

Blot: S

Whole cell extract

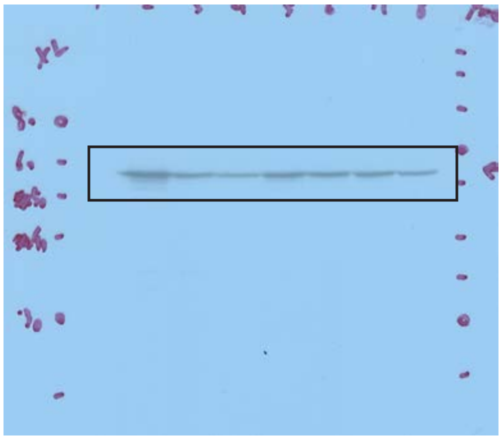

Blot: Myc

Supplementary Fig. S14 (original data of Figure 5f)

Pull-down: S-Agarose

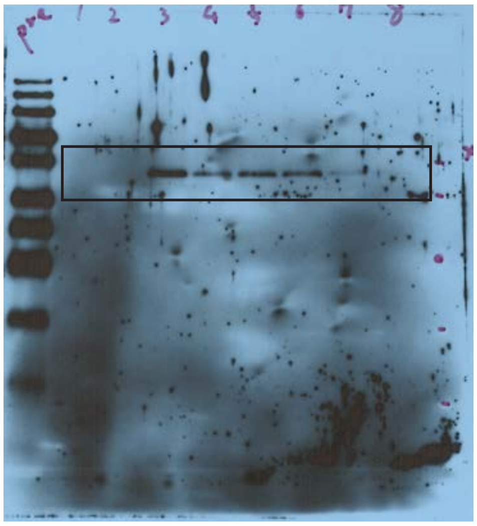

Blot: FLAG

Pull-down: S-Agarose

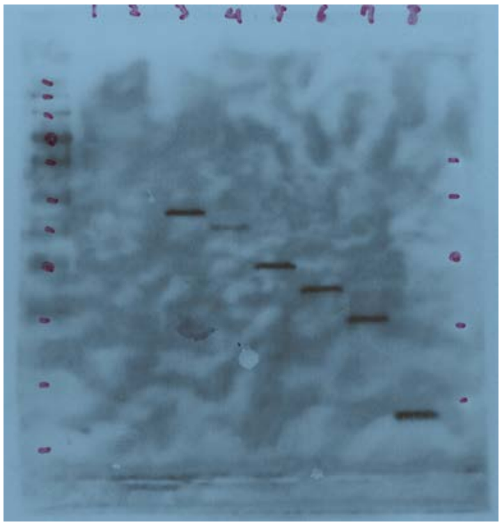

Blot: S

Whole cell extract

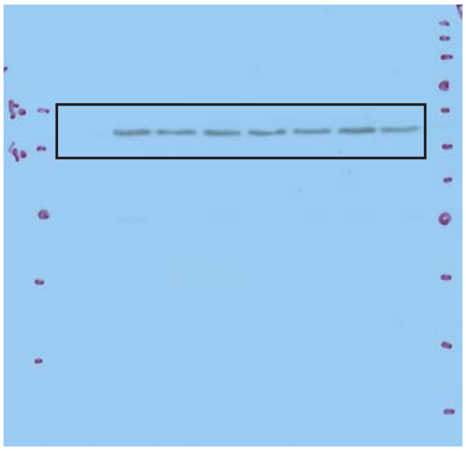

Blot: FLAG
